# Clinical and Radiological Study of Serum Fluoride in Relation to Knee Osteoarthritis

**DOI:** 10.5704/MOJ.2011.023

**Published:** 2020-11

**Authors:** VK Singh, KS Rathore, G Khan, A Rahim, A Rashid, S Chauhan

**Affiliations:** Department of Orthopaedics, NIMS Medical College and Hospital, Jaipur, India

**Keywords:** fluorosis, knee, arthritis

## Abstract

**Introduction:**

Fluorosis has been associated with an increased risk of degenerative changes in the knee. Multiple studies have found an association between arthritis and elevated fluoride levels. We aim to delineate if elevated fluoride level has any direct correlation with the degree of radiological grading and clinical symptoms in knee arthritis.

**Materials and Methods:**

A cross-sectional study of 80 knee arthritis patients was conducted from February 2017 to April 2018. Serum fluoride levels were measured and patient’s pain scores, WOMAC scores and radiological grading were correlated with the elevated fluoride levels.

**Results:**

In our study, 30 out of 80 patients had increased serum fluoride level. Statistically significant differences were noted in VAS score, WOMAC score and Kellgren and Lawrence radiological grades between patients with normal serum fluoride level and those with elevated fluoride level.

**Conclusion:**

There is an increased risk of knee arthritis in patients with elevated blood fluoride levels and patients with increased fluoride levels are associated with more severe symptoms and radiographic disease.

## Introduction

Osteoarthritis (OA) is the most common joint disease of mankind and is also the leading cause of chronic disability in developed countries^1^. Osteoarthritis is the second most common rheumatologic problem and it is the most frequent joint disease with a prevalence of 22% to 39% in India. It is more common in women than men, but the prevalence increases dramatically with age. Nearly 45% of women over the age of 65 years have symptoms while radiological evidence is found in 70% of those over 65 years. It is a slowly progressive joint disease, characterised by osteophyte formation, cyst formation, subchondral sclerosis, mild synovitis, capsular fibrosis and various morphological and biochemical changes in joints^2^.

OA has multi-factorial aetiologies and occurs due to an interplay between systemic and local factors. Fluorosis can expedite joint wear and tear and is supposed to be one of the important factors contributing to knee arthritis^3^. Fluorosis is a disease caused by an excess of intake of fluoride, usually by drinking water. It is endemic in 22 countries of the world including India. In India, 19 states with 275 districts out of a total of 619 have been identified as endemic areas for fluorosis, with an estimated 25 million people impacted, and another 66 million at risk. Endemic fluorosis presents commonly as dental fluorosis in children, skeletal fluorosis in adults, and non-skeletal fluorosis. Recent research clearly shows that fluoride-induced joint pains can occur in the absence of obvious skeletal fluorosis. This makes fluoride’s effects on joints extremely difficult to differentiate from common forms of arthritis. In fact, research has found that fluoride can be a direct cause of osteoarthritis, with or without the presence of classic skeletal fluorosis. Debilitating knee pain due to knee arthritis requires long-term treatment which has a huge economic burden^4^. Pain, when severe, requires knee arthroplasty which is an expensive surgery. Patients in the developed world either have health insurance or have a good national health system which pays for the treatment cost. Knee osteoarthritis is rampant in India and when it affects patients of poor socioeconomic class, it has devastating consequences. This cohort of patients have no access to knee arthroplasty because of financial reasons and they have no choice but to suffer in pain. A lot of work has already been done on knee osteoarthritis and fluorosis but there is no literature correlating the severity of knee osteoarthritis with serum fluoride levels.

## Materials and Methods

A cross sectional study was conducted at NIMS super-specialty hospital from February 2017 to April 2018 after ethical committee approval. A pilot study was done on 12 patients to calculate the sample size for 80% power with α error of 0.05, assuming correlation coefficient (ρ) as 0.310 between serum fluoride level and WOMAC scoring system among knee osteoarthritis patients. This pilot study showed that 80 patients were necessary for an 80% power.

Males or females between 40 to 60 years who consented to participate were included in the study. We excluded patients outside this age range, patients with history of trauma, infections or any other systemic disorders like diabetes, tuberculosis, or any other long-standing illnesses or any inflammatory conditions like rheumatoid arthritis. We did not include patients below 40 years as they would not meet the ACR guidelines on the diagnostic criteria of knee osteoarthritis. On the other hand, an age above 60 years would confound the study as WHO reported a rise in the incidence of primary osteoarthritis to as much as 10% in the general population of that age group. Our hospital caters a large number of rural populations from surrounding districts where fluorosis is endemic.

Patients were divided into two groups based on normal or elevated fluoride levels. Group A comprised of 30 patients who had normal serum fluoride levels. Group B had 50 patients who had elevated serum fluoride levels.

To remove any selection bias, 80 consecutive patients who satisfied inclusion criteria were enrolled in the study. In the case of patients with bilateral knee pain, the more severely affected knee was included in the study. All patients who were included in study were squatters and were routinely involved in squatting during day to day activities. After enrolment, a detailed history and full clinical examination was performed. Pain was graded on VAS scale. WOMAC scoring system was used for assessing pain, stiffness and physical function. A standing antero-posterior and lateral radiograph of knee was taken and radiological grading was done using Kellgren and Lawrence grading system. Blood samples were withdrawn for complete blood counts, erythrocyte sedimentation rate, blood sugar, serum uric acid, rheumatoid factor and serum fluoride level estimation. Serum fluoride level was estimated by using a special fluoride ion specific electrode [Thermofischer scientific, Orion 4 star bench top pH/ISE metre, Singapore]. Serum fluoride levels more than 0.30 mg/litres were considered as high fluoride levels. SPSS, version 19.0 [IBM Corp., Armonk, NY, USA] was used for all statistical analyses.

## Results

Group A had 22 males and 8 females while Group B had 38 males and 12 females. Male to female ratio were comparable between two groups. Average age of patients in Group A was 55.4 (Range 42-60) and 56.2 (Range 45-60) in Group B. There was no statistical difference between the two groups. The mean VAS of patients in Group A was 6.56 and that of Group B was 7.26, which was statistically significant (p value, 0.023, Table I). The mean WOMAC Score of patients in Group A was 47.63 and that in Group B was 54.34.

**Table I:** Comparison of VAS score with serum fluoride level.

Serum Fluoride Level	n	Mean	S.D.	p value*
Normal	50	6.56	1.215	0.023
Increased	30	7.267	1.081	

*Mann-Whitney Rank Sum Test

We also evaluated the three sub-components of WOMAC i.e. pain, stiffness, and function individually to compare results between patients with normal and elevated fluoride levels. The mean WOMAC pain score of patients in Group A was 8.82 and that in Group B was 10.33, which were statistically significant (p value, 0.037, Table II). The mean WOMAC stiffness score of patients in Group A was 4.16 and that in Group B was 4.83, which were statistically not significant (p value, 0.076, Table II). Finally, the mean WOMAC physical function score in Group A was 32.74 and that in Group B was 36, which was statistically highly significant (p value, 0.002, Table II). The mean Kellgren and Lawrence grading for Osteoarthritis in Group A was 2.38, and in Group B was 2.90, which was statistically significant (p value, 0.012, Table III).

**Table II:** Comparison of WOMAC score with serum fluoride level

WOMAC SCORE	Serum Fluoride Levels	n	Mean	S.D.	p* value
Pain subscale	normal	50	8.82	2.746	0.037
	increased	30	10.33	2.617	
Stiffness subscale	normal	50	4.16	1.346	0.076
	increased	30	4.833	1.555	
Physical function	normal	50	32.74	4.956	0.002
	increased	30	36.8	5.149	
Total	normal	50	47.63	8.735	0.004
	increased	30	54.34	9.011	

*Mann-Whitney Rank Sum Test

**Table III:** Comparison of Kellgrens and Lawrence grades with serum fluoride levels

Serum Fluoride Level	n	Mean	S.D.	p* value
Normal	50	2.38	0.753	0.012
Increased	30	2.9	0.803	

*Mann-Whitney Rank Sum Test

## Discussion

Osteoarthritis of the knee is extremely common in India. Age, female gender, increased body weight and trauma are some of the common risk factors associated with knee osteoarthritis^4^. Understanding the risk factors and their modification may reduce the risk, prevent disease progression and reduce pain and associated disability^5^.

Pinet *et al* explained in detail about fluoride-induced arthritic changes in spine, elaborating lipping and syndesmophyte formation in the spine due to elevated fluoride levels^6^. Waldbott in his study showed that excess fluoride leads to changes which are similar to osteoarthritis. There is formation of apatite microcrystals due to high fluoride which leads to calcification of ligaments and joint capsules^7^. In our study, patients with elevated fluoride level showed a higher degree of osteoarthritis radiologically as per Kellgren and Lawrence grading.

Tartatovskaya concluded that fluoride exposure could exacerbate the degenerative effect of physical stress on joints, with or without the presence of radiologically detectable skeletal fluorosis^8^. Savas *et al* in 2001 found strong evidence of a fluoride-osteoarthritis link in individuals who did not have tell-tale sign of skeletal fluorosis^9^. Hileman reported that because the clinical symptoms mimic arthritis, the early clinical phases of skeletal fluorosis is easily misdiagnosed^10^. Hodge and Smith showed the presence of ectopic calcification in cartilage in fluorosis patients^11^. Sharma studied the effect of fluoride on collagen and reported that sodium fluoride produces abnormal collagen fibres which provide an abnormal environment for calcification^12^. Sharma in another study concluded that fluoride, ingested in excessive amounts, increases the solubility and degradation of collagen and reduces the collagen biosynthesis and cross-links. Therefore, the matured tissue collagen fibers would be abnormal due to inadequate cross-linking^13^. Srivastava *et al* concluded that fluoride causes breakdown of hydroxyproline which is responsible for stabilisation of collagen triple helix^14^. Thus, destruction of collagen in the cartilage occurs. An abnormal growth of the bone and the cartilage also occurs as the collagen-producing cells try to compensate by producing larger quantities of imperfect collagen.

Ji *et al* mentioned that fluorosis has a close connection with the metabolism of cartilage tissue and excess fuoride can damage the cartilage^15^. In our study, patients with higher fluoride level were associated with more severe disease both clinically and radiologically. Our study findings are in line with the study of previous authors that high fluoride and arthritis are directly related. This would also explain the high incidence of knee osteoarthritis in areas endemic for fluorosis. Authors would recommend that safe drinking water in endemic areas will most definitely decrease the burden of knee arthritis and reduce the disabilities associated with it.

Our study is limited by small numbers but the findings of study clearly demonstrate that high fluoride levels are associated with an increased risk of knee arthritis. We tried to eliminate the confounding factors which could affect the results. All patients in both groups were routinely involved in squatting in their day to day activities. Both groups were comparable in terms of male female ratio as well as age. We did not compare BMI of both groups as all patients included in the study were from rural areas and incidence of obesity in rural population is negligible.

## Conclusions

Our study demonstrated that there is an increased risk of knee arthritis in patients with elevated serum fluoride levels. Patients with increased fluoride levels are associated with more severe symptoms and radiographic disease. Fluorosis is a big menace to its endemic population and safer drinking water will reduce economic burden due to knee arthritis.
